# A Study of the Best Means of Local Anesthesia for Extraction of Teeth

**Published:** 1904-11

**Authors:** E. Sauvez

**Affiliations:** Paris, France.


### A STUDY OF THE BEST MEANS OF LOCAL ANESTHESIA FOR EXTRACTION OF TEETH.

By Dr. E. Sauvez, Paris, France.

(*Abstract of paper read at the Fourth International Dental Congress, St. Louis, 1904.*)

We should like to plead especially in favor of local anesthesia, in which we have absolute confidence, thoroughly persuaded that we speak in the interests of the patients and of the dentists and feeling assured that the operations will be thereby but the better executed.

General anesthetics act by inducing functional arrest in the nervous centers, according to an immutable, hierarchical sequence, in the first place, in the tégument, then in the medular, reflex centers, and finally in the bulb; if anesthesia is still further developed, the bulbous centers become paralyzed and death ensues.

According to the *Edinburg Medical Journal,* of November, 1903, the ratio of mortality produced by general anesthesia is as follows:

Chlorure ethyl—1/16.000
Bromure—1/4.000
Ether—1/12.000
Choloroform—1/2.000
Photoxyde azote—Impossible of calculation.

However, 13 cases of death due to protoxyde are known (cases of Maurice Perrin, of Magitot, of Watson and of Duchesne.)

And we are dealing only with fatal cases. We do not mention the frequent and serious accidents, the blue syncopes from which the patients are only saved by artificial respiration. According to the witty expression of Professor Recus, "for an accident to count, it must be fatal."

We show in our memoir the difficulties of operation due to the inertia of the completely anesthetized patient and to the decubus dorsal, or reclining position, in which he is generally placed.

In case of fatal accident, when the matter is brought to court, the judges, who would never question the use of an anesthetic for a serious operation, will often bitterly contest its employment when it is used merely for the extraction of a tooth.

After citing all the substances abandoned as impracticable, for instance, tincture of cannabis indica, strong carbolic acid, orthoforme, etc., we lay stress on the use of electricity as an agent of local anesthesia.

Electricity has been employed in two ways:

*A*) A current of high frequence directed towards the level of a tooth during a variable period previous to extraction (Regner and Didsbury).

*B*) The introduction of medicinal substances (cocaine) within the tissues, by the aid of electricity. This is the cataphoresis based on the theory of Faraday's ions (Pont).

After expounding the operating manual of these means of local anesthesia, we develop its critique and show that electricity, as an agent of anesthesia, is not to be depended upon, presenting many inconveniences and difficulties.

We are thus forced to bring forward cocaine, or its derivatives, which, in injections, seem to give practical results superior to all other methods of local anesthesia known at present.

Practically, cocaine is a local anesthetic; from a physiological standpoint, it is a general as wel l as a local anesthetic. * * * Injected into animals in physiological doses, cocaine determines in them an extreme muscular excitation, followed by an insensibility exclusively of the surface, the deeper sensibility being preserved.

One of the most remarkable of the properties of cocaine is its vaso-constrictive action. We emphasize this fact and expose its divers consequences. Let us here only cite the elevation of the blood pressure. We dwell as well on its action on the centers of thermic regulation (cocaine heightens the temperature, Richet) and on the ocular apparatus (Mydriase).

We now approach the dangers which the use of cocaine might offer. In the first place, there is syncope. We show its rarity and explain how easy it is to avoid it by employing the right dose on the reclining patient. The other phenomena observed, sometimes subsequent even to correct injections of cocaine, are slight and insignificant—slight tingling (pins and needles) of the extremities and greater loquacity. There is really nothing in local anesthesia by cocaine approaching the sudden frights which accompany the use of chloroform. Statistics bear us out in this statement. In 7,000 cases of anesthesia by cocaine practised by Reclus, he has not noted the slightest trouble in the physiological equlibrium. We ourselves, with a minimum of 15,000 injections, cannot register one accident not even an incident, due to the use of cocaine. We are, therefore, in a position to affirm that this is the most inoffensive of all the anesthetics and that it exposes one to no surprise whatsoever.

With the aim of avoiding the so-called inconveniences attributed to cocaine, its derivatives or congeneric substances have been experimented with, which subject is treated at length in our thesis. We will here limit ourselves to citing the conclusions we arrived at concerning tropacocaine and eucaine.

Tropacocaine presents an equal degree of toxicity while its anesthetic action appears less profound than that of cocaine.

Eucaine is a vaso-dilator, its injection is painful and it presents a feebler power of anesthesia and of a shorter period of duration than cocaine.

Phenate of cocaine, insoluble in water, and employed, therefore, dissolved in oil or liquid vaseline, produces nodules which are long in disappearing.

Therefore the use of chlorhydrate of cocaine appears to us to carry with it all the advantages claimed for the preparations compared to it as much from the point of view of the anesthesia produced as by the percentage of possible accidents.

(The author reports experiments with various vehicles for carrying the cocaine, such as oil, vaseline, cocoa-butter, etc., and reports adversely.)

Because of the reasons we have given, it is water we choose as vehicle for chlorhydrate of cocaine. It remains to determine what title to give this solution. Cocaine being a toxic medicament, one should use as small a dose as possible, in spite of the fact of its being endowed with sufficient analgesic power. There is general agreement today to recognize a 1 part to a 10[illegible] solution as sufficient.

The toxicity of cocaine depends, not only on the weight of the quantity of the injected alkaloid, but also on the quantity of water in which it is in solution. The greater the quantity of water used, the more inoffensive it is for the same amount of cocaine.

For the extraction of a tooth, one cubic centimeter of a 1 part to a 100 solution—that is to say, one centigramme of cocaine quite suffices.

Schleich employs even a feebler solution: 0 gr. 002 and even 0 gramme 001. But in this case, the anesthetic is illusionary and due simply to the mechanical distension of the tissues; similar results can be obtained by a simple injection of distilled water.

Ordinarily for the extraction of a tooth we use one centigramme of cocaine. Under 12 years and above 60, we reduce the dose to no more than one-half centigramme. We have never had an accident and have yet always produced perfect anesthesia. In certain cases of complicated extractions, we inject as high as 2 and 3 centigrammes without accident, but conform, of course, to the precautions stated above.

We make use of fresh solutions, or extemporaneous solutions or of solutions preserved in sterilized ampoules.

We conclude that a cubic centimeter of a fresh and sterile solution of chlorhydrate of cocaine, one part to a hundred of distilled water, seems sufficient for practice in the majority of cases, and such a dose induces neither accident nor incident.

We lay stress on the precautions to be observed—the loosening of any clothes which might in any way impede respiration, a horizontal position, or, at any rate, one approaching the horizontal. There are contra-indications; as cocaine raises the blood pressure, it is contra-indicated for aortics and arteriosclerous patients.

Because of its depressive action, it ought also to be forbidden for anemic individuals and those debilitated, extreme nervous or averred neuropaths, and those worn out with debilitating diseases. Should the patient present a general state which would seem to visibly predispose him to syncope, it were better to abstain. These contra-indications are not absolute, they are but relative, and the more to be observed as the disease seems to arrive at a more serious degree.

As the gums offer quite a resistance to the injection, it is well to use a Prav[illegible] syringe with a needle screwed on. Stee[illegible] needles, by reason of their rigidity and th[illegible] delicacy of their vent are preferable.

A great many of the accidents of infection attributed to cocaine are really due to aseptic faults. We keep our syringes in a carbolic solution (5 p. 100) with the pi[illegible] ton drawn out, so as to keep the body of the pump and the piston-rod constantly i[illegible] contact with the antiseptic liquid. The needles are to be boiled at least five minutes, or held in the flame.

The mouth should be rinsed with boracic acid and water, the gum washed with alcohol.

As the first puncture is liable to be somewhat painful, even with fine needles, we apply to the gum previously dried, [illegible] 10 p. 100 solution of cocaine, or else pulverized coryl.

After exposing the character of the tissues to be reached by the injection, we arrive at the following conclusions:

To insure the efficacy of the injection, it must be made at the level of the mucous membrane, which adheres closely to [illegible] periostium, and consequently not too near the neck.

The syringe, armed with the needle, is held like a pen and forced into the gu[illegible] not deeply, within the derma, at a point situated about equidistant between the free edge of the gum and the presumed spot where the point of the root should be obliquely in reference to the median region of the maxillary. A sufficient resistance is felt when injecting and bit by bit the mucous membrane whitens under the influence of the cocaine.

One can be sure that the anesthesia will be excellent if the piston pushes hard. If the liquid enters without resistance, it shows that the injection has been made in the cellular tissue and the formation of an oedemous bulb will be determined. It were better then to withdraw the needle and begin again. Several injections are necessary, at least two, in order to surround the tooth with an anesthetized zone.

Adrenaline added to cocaine has given the best results, from the local as well as from the general point of view. (Batlin and de Nevreze.)

Stovaine is a substance recently discovered by a French chemist, M. Fourneau, which offers certain advantages over cocaine.

1. Weaker toxicity. From experiments on rabbits and guinea pigs which we describe in our paper, it is shown that this difference of toxicity between the two substances is very great—in fact, cocaine is shown to possess twice the toxicity of stovaine.

2. That this new medicament possesses a vaso-dilatative action, while cocaine's action is vaso-constrictive. Consequently with stovaine a patient may be operated upon, seated.

3. Finally, stovaine costs very much less than cocaine.

It is about two months ago since we began using stovaine—that is to say about the end of January, 1904, upon the advice of Professor Reclus. This surgeon has been employing it for several months in his service, and has obtained perfect results up to the present.

Personally, we have made a hundred injections of a 0.75 p. 100 solution of stovaine. We have observed no menace of syncope, no sickness ensues and we can affirm that the anesthesia produced is equal to that produced by cocaine.

1. Complete anesthesia, because of the dangers and inconveniences it entails, should be the exception in dental surgery. On this fact is based the importance of local anesthesia.

2. Of all the methods of local anesthesia actually known, cocaine seems to give the best practical results.

3. Chlorhydrate of cocaine appears to us superior to all the other preparations which compete with it.

4. Distilled water is the best vehicle proposed for the transmission of cocaine.

5. In general practice, a satisfactory anesthetic is obtained and all accident is avoided by the use of one cubic centimeter of a fresh solution, in distilled water, of chlorhydrate of cocaine at 1 p. 100.

6. When the injection exceeds one centigramme of cocaine, a horizontal position is called for.

7. In the extraction of a tooth, it is almost exclusively the rending of the alveolo-dental ligament that causes the pain.

8. The anesthesia is entirely dependent upon the manner of operating the injection. The after effects depend on the asepsis.

9. After the operation the patient should remain reclining one-quarter of an hour for one centigramme of cocaine; from two to three hours for a larger dose. --*Items of Interest.*

